# AQUA^©^ as predictor of allergy in elite marathon runners

**DOI:** 10.1186/1939-4551-7-7

**Published:** 2014-04-03

**Authors:** Renata N Teixeira, Felipe AR Mendes, Milton A Martins, Timothy D Mickleborough, Celso RF Carvalho

**Affiliations:** 1 Department of Physical Therapy, School of Medicine, University of Sao Paulo, Av. Dr Arnaldo, 455, Room 1210, 01246-903 São Paulo, SP, Brazil; 2 Department of Medicine, School of Medicine, University of São Paulo, Av. Dr. Arnaldo 455 – room 1210, 01246-903 São Paulo, SP, Brazil; 3 Department of Kinesiology, Indiana University, 1025 E. 7th Street, HPER 112, Bloomington, IN 47401, USA

## Abstract

**Background:**

The prevalence of allergy in athletes is increasing, and its risk varies across sports. The risk is dependent mainly on the ventilation rate and environmental factors; however, the prevalence of allergy in elite runners remains unknown. Therefore, the aim of this study was to screen allergy symptoms in elite marathon runners by using a validated questionnaire for screening allergy in athletes.

**Methods:**

Two hundred and one elite marathoners, who participated in the most competitive Brazilian marathons and half-marathons during 2011, were invited to complete a validated self-report Allergy Questionnaire for Athletes (AQUA^©^), with additional questions pertaining to training history, such as running experience, running distance per week and their best race time in marathon or half-marathon events.

**Results:**

Sixty percent of the assessed athletes reported allergy symptoms as defined by a positive AQUA outcome (score [greater than or equal to] 5). No significant differences (p > 0.05) between groups (AQUA + and AQUA-) were observed for gender, age, running experience, weekly training volume and best performance time in the half-marathon and marathon. The most frequently reported symptoms were related to the respiratory tract and physical effort.

**Conclusions:**

This study demonstrates that AQUA^©^ can be used to predict allergy in elite marathon runners. In addition, these athletes have a higher prevalence of allergy symptoms to elite athletes from other sports.

## Introduction

Allergy disease is a common condition which affects more than one out of five individuals, and is characterized by an abnormal response of the immune system to harmless substances such as pollen, dust, animal dander, food, drug and venom [1]. The prevalence of allergic diseases in elite athletes has been reported to range from 10% to 74% [2-4], and this wide variability appears to depend on the diagnostic method used to quantify allergic symptoms, the environmental conditions, an athletes’ training history (i.e. intensity, frequency and volume) and sport modality [5-7]. Despite studies showing high prevalence rates of allergic symptoms in athletes [8,9], no specific diagnostic protocol has yet been established as part of a routine clinical assessment in this cohort [3,10,11]. Bonini and colleagues have developed the Allergy Questionnaire for Athletes (AQUA^©^), which has been validated, and has a high predictive value, for identifying athletes with allergic airway disease [6]. Allergy screening utilizing the AQUA^©^ questionnaire has been previously used in soccer players [6], Olympic athletes [12], and recreational runners [7].

It has been shown that high-performance athletes, especially those who take part in winter or high-ventilation sports have a higher prevalence of allergic symptoms [5,13,14], and are therefore more susceptible to allergic illnesses due to an association between the repeated physical stress of intense training, and frequent exposure to unfavorable environmental conditions, such as high levels of aeroallergens, pollution, pollen, and changes in air temperature (i.e., dry and cold air) [2,15].

Long distance running (i.e. marathon) is an endurance sport that requires an extensive amount of training, especially at the elite level, and consists of a high running volume, in conjunction with high-intensity continuous running and short bursts of high-intensity efforts. As such there is evidence to suggest that high-volume and high-intensity training may cause overtraining leading to immunosuppression and increased susceptibility to allergic diseases [16].

To the best of our knowledge, there is no data regarding the prevalence of allergy symptoms in elite marathon runners in Brazil. Therefore, the aim of this study was to screen for allergic symptoms in elite marathon runners using the AQUA^©^ questionnaire.

## Methods

### Subjects

Two hundred and thirty elite marathon runners were invited to take part in this cross sectional study. The athletes were recruited from elite Brazilian marathon and half-marathon runners who competed during the 2011. The inclusion criteria were: age between 20 and 50 years, and completion of a marathon in the past 18 months under a time of 2 hr:35 min:00 sec for men and 3 hr:00 min:00 sec for women, or a half-marathon time below 1 hr:23 min:00 sec for men and 1 hr:35 min:00 sec for women; this criteria was adopted to ensure that we recruited completive endurance runners. All of the athletes who agreed to participate in the present study gave written informed consent prior to enrollment in the study. The clinical protocol was approved by the local human research ethics committee.

### Study design

Allergy symptoms were evaluated by the AQUA^©^[6], a validated questionnaire specifically developed for athletes, which has been translated into 9 European languages, including Portuguese. The permission to use the AQUA^©^ was granted by the copyright holders. The AQUA^©^ has previously been validated in 128 professional football players [6], in conjunction with the diagnosis of allergy using salivary immunoglobulin E determination and skin prick testing, and detailed allergy history. A mean total AQUA© score of greater than five has the best positive predictive value for allergy (0.94), with a sensitivity of 58.3% and specificity of 97.1% [6]. The AQUA^©^ consists of 25 items regarding allergic symptoms, family history of allergy, suspicion of allergy, and the use of allergy medicines. In the present study, questions 4 to 16 (except number 14) received a score, and the sum of these questions was used to classify AQUA + athletes (presenting a score ≥5), and AQUA- athletes (presenting a score <5), as previously described [6]. Athletes were asked additional questions about running experience, running distance per week, and their completive performance in marathon and half-marathon events.

### Data analysis

The data were analyzed using the Sigma Stat 3.5 statistical package (Chicago, IL). The Kolmogorov-Smirnov test was used to verify the normality of the data, and Levene’s test was used to test for homogeneity of variance between groups. The Student’s t-test and the Chi-squared test were used to compare continuous and categorical data between groups (AQUA + and AQUA- athletes), and difference between genders. The data are expressed as the median (non-parametric) or mean (parametric) with a 95% confidence interval (95% CI). The level of significance was set at p ≤ 0.05.

## Results

Twenty-nine out of 230 invited athletes declined to participate in the study. The average age was 34 years old (22 to 49 years, 95% CI), with no significant difference being observed between males (22 to 50, 95% CI) and females (22 to 48, 95% CI) for average age. The athletes training history, and racing experience and completive performance are shown in Table 1. The mean total AQUA^©^ score for the AQUA + athletes was 12.5 ± 6.3 (range, 5–27).

**Table 1:** Characteristic of athletes’ training, experience and performance

***Characteristics***	***All athletes***	***Male***	***Female***
	***(n = 201)***	***(n = 165)***	***(n = 36)***
*Training sessions (n/week)*	6.7 (6–7)	6.7 (6–7)	6.8 (6–7)
*Running distance (km/week)*	180 (140–220)	180 (150–220)	160 (120–217)
*Running experience (years)*	11 (4–30)	11 (3–30)	11.5 (4–26)
*Performance time in 21 km (min)*	70 (62–87)	68 (62–80)	79 (73–91)
*Performance time in 42 km (min)*	148 (132–170)	143 (132–155)	160 (137–176)

Legend: data are presented as mean and 95% confidence interval (in parenthesis); min = minutes, km = kilometers.

There was no significant difference between groups (p > 0.05) (AQUA + *vs.* AQUA-) in terms of gender, age, running experience, weekly training volume, and best performance time for the half-marathon and marathon (Table 2). Of the 201 athletes analyzed, 122 of the athletes (60.7%) presented an AQUA + score ≥5), while 79 of the athletes (39.3%) presented an AQUA- score <5.

**Table 2:** Anthropometric and training data between AQUA + and AQUA- athletes

	***AQUA+***	***AQUA-***	***P = value***
	***(n = 122)***	***(n = 79)***	
*Gender (F/M)*	26/96	10/69	NS
*Age (years)*	33 (22.0-48.4)	34 (23.4-49.0)	NS
*Running experience (years)*	12.0 (4.0-28.2)	10.0 (3.0-29.5)	NS
*Running distance (km/week)*	180.0 (140.0-220.0)	180.0 (140.0-234.0)	NS
*Performance time in 21 km (min)*	70.0 (63.0-88.0)	70.0 (62.9-84.0)	NS
*Performance time in 42 km (min)*	149 (132–168)	149 (134–168)	NS

Legend: data are presented as mean and 95% confidence interval (in parenthesis), except for gender expressed in absolute number. F = female, M = male, min = minutes, km/week = kilometers per week; NS = non-significant.

**Figure 1:**
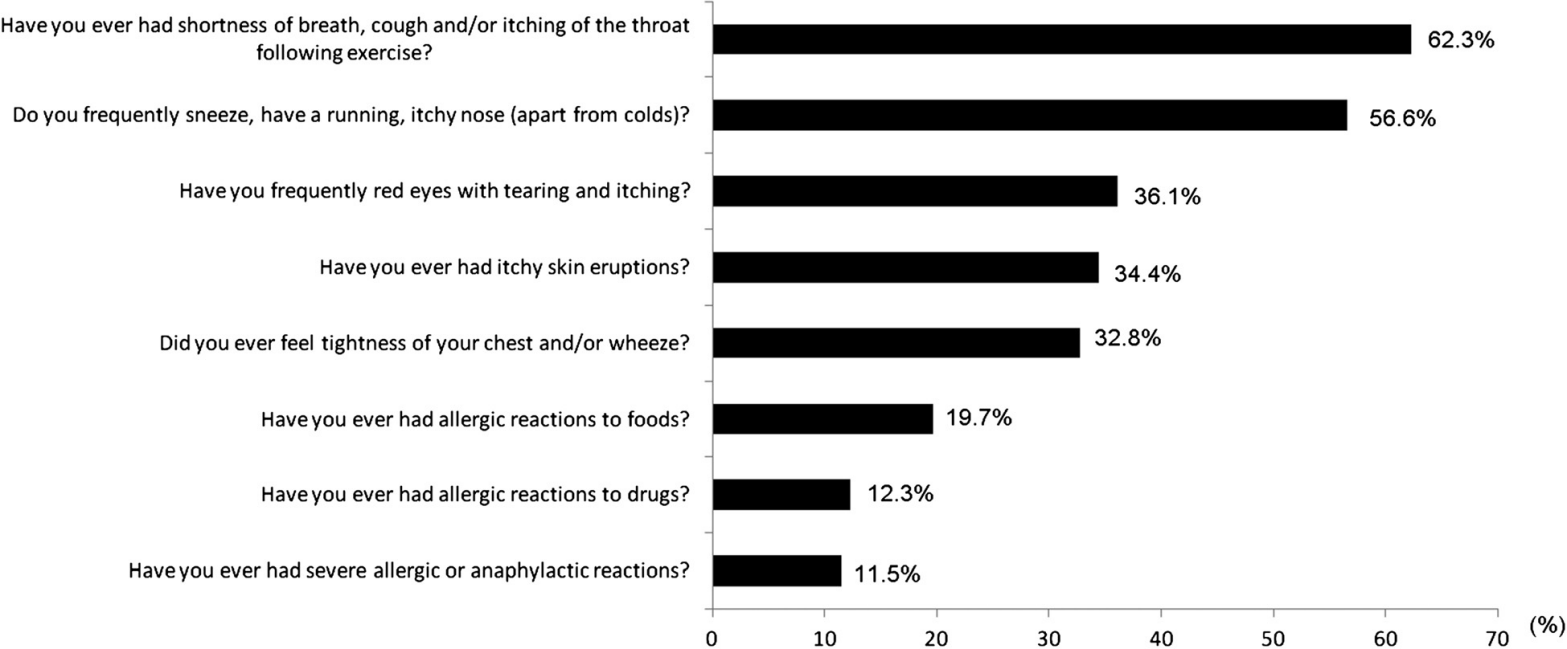
**Frequency of the most reported AQUA**^**© **^**questions in athletes with AQUA+ score.**

Among the AQUA + athletes, 33 (25%) athletes reported having only one allergic symptom, while 36 (30%) had two, and 55 (45%) had three or more. Fifty-six (46%) of the AQUA + athletes reported having physician-diagnosed allergy, 74 (60.7%) were suspected of suffering from allergy, and 46 (37.7%) reported using, or had used, anti-allergy drugs. The proportion of positive answers for the most frequent questions is shown in Figure 1.

## Discussion

To our knowledge the present study is the first to show that elite marathon runners have a high prevalence of allergic symptoms independent of gender, age, quantity of training and performance times. Based on the data from the present study, we suggest that an allergy screening test should be included as part of the clinical assessment of endurance and high-level athletes.

We found that 60.7% of the subjects presented with allergy symptoms as defined by a positive AQUA^©^ questionnaire score. In the validation study of the AQUA^©^ questionnaire, Bonini et al. assessed 128 professional soccer players [6] and observed an allergy prevalence of 32%, approximately half the rate observed in the present study. The discrepancy in the prevalence rate for allergy between our study and the study of Bonini et al. [6] study may be due the fact that elite marathon runners maintain higher ventilation rates for a longer periods of time, and this inhale greater amounts of allergens and airway irritants compared to soccer players, who experience short bursts of high-intensity exercise.

In the present study the majority of the reported symptoms were related to the upper respiratory tract, with exercise-induced bronchoconstriction (EIB) being the most prevalent reported symptom. Thus, 62% of the athletes considered allergic (AQUA+) claimed shortness of breath, cough and/or itchy throat during or following exercise (AQUA^©^ question 13). The prevalence of EIB symptoms was higher than those observed in soccer players and recreational runners (11.7% and 32%, respectively) [6,7], and was even higher than in a study previously performed by our group that assessed the prevalence of EIB, using eucapnic voluntary hyperventilation (EVH), in elite long distance runners [17]. However, the lack of an objective assessment, such as EVH, may have influenced the higher prevalence of allergic symptoms that were observed in the present study compared to our previous study [17].

It should be noted that the AQUA^©^ was previously validated against skin tests and has a high specificity (97.1%) but a low sensitivity (58.3%) [6]; it is quite possible that AQUA + group included subjects with respiratory allergy and other “allergic” symptoms, and that not all AQUA + subjects have necessarily to be skin test positive. We also observed that 56.6% of our athletes reported allergic rhinitis, which was the second most prevalent respiratory symptom. The prevalence of rhinitis in our population seems relatively high, as it is more than the levels observed in German elite athletes [11]. There are three possible explanations for the higher prevalence rate of respiratory symptoms observed in the elite marathon runners in the present study compared to other athletes [11]: i) previous studies [6,11] have included athletes from low- to moderate exercise intensity sports, and thus less likely to trigger allergic symptoms ii) high performance marathon runners shift from nasal to mouth breathing as a consequence of high-intensity training, and this shift induces airway drying and cooling, and contributes to airway epithelial damage [18,19]; and iii) the physical demands of elite marathon runners may outweigh the body’s ability to fully recover between training sessions and competitions, reducing the athletes immunological response and predisposing them to upper airway infections [20].

Environmental factors may also aggravate the effect of heavy training on the airways of athletes [21]. For instance, there are many environmental conditions that are sport specific and can induce adverse effects on the respiratory system, such as pollen exposure in athletes who train outdoors, cold air in winter athletes [21,22], fine particles in ice rinks [23] and chlorine exposure in swimmers [24]. In our study, we compared the prevalence of allergy between elite marathon runners living in metropolitan and rural areas, and did not observe any difference between these two environments (data not shown). Moreover, in our study, environmental conditions do not seem to explain the high prevalence of allergy; however, since we did not perform skin tests, it is possible that environmental factors may be more relevant in subjects with symptoms, but with negative skin tests (for instance in EIB in non-allergic subjects). Thus, further studies are required to investigate whether environmental conditions affect the prevalence of allergy in elite marathon runners.

A limitation of this study was that we did not assess blood levels of immunoglobulin E (IgE) to quantify allergy. However, it seemed unnecessary because the AQUA^©^ questionnaire has been previously validated based on its association with IgE levels, and demonstrating high specificity [6]. Moreover, according to our previous experience with elite runners [17], blood collection is not well accepted either by athletes or by their coaches due to possible doping concerns.

## Conclusion

This study demonstrates that AQUA^©^ can be used to predict allergy in elite marathon runners. In addition, these athletes have a higher prevalence of allergy symptoms to elite athletes from other sports. We also suggest that the AQUA^©^ may be a useful tool in the routine examination to identify athletes with allergy symptoms, in conjunction with accurate allergy testing and functional assessment.

## Competing interests

The authors declare that they have no competing interests.

## Authors’ contributions

RNT have made substantial contributions to the acquisition of data, design of the study and draft of the manuscript. FARM performed the statistical analysis and helped to the draft manuscript. MAM made contributions to conception and design of the study and helped to draft the manuscript. TDM was involved in revising the manuscript for intellectual content. CRFC participated in the study design and coordination and helped to draft the manuscript. All authors read and approved the final manuscript.
